# Metabolic/bariatric clinical practice in Lagos, Nigeria: a retrospective review of the cases and outcomes of our 10-year experience

**DOI:** 10.11604/pamj.2025.51.67.45052

**Published:** 2025-07-04

**Authors:** Abuchi Okaro

**Affiliations:** 1 Euracare Multi-Specialist Hospital, 293 Younis Bashorun Street Victoria Island, Lagos, Nigeria

**Keywords:** Bariatric surgery, obesity, metabolic surgery, diabetes

## Abstract

Obesity is recognized as a major public health hazard, as it is linked to multiple chronic diseases. Globally, metabolic or bariatric surgery has been proven to be a safe and effective treatment for obesity, yet access to metabolic or bariatric surgery in Nigeria, West Africa continues to be limited. This study reviews the ten-year experience of an individual bariatric surgeon and his team in Nigeria. The patient database was used to collate and record all patient specific information. This database was then accessed to extract required information on patients treated in our specialist clinics between January 2012 and June 2022. There were a total of 381 consecutive patients. The mean age was 41.4 years (+/- 9.1). Females made up 314 (82.4%) in the cohort. Mean initial body mass index (BMI) was 42.4kg/m^2^ (+/- 8.7). 220 (57.7%) had endoscopic intra-gastric balloon placement, 157 (41.3%) underwent laparoscopic sleeve gastrectomy, and 4 (1%) patients underwent laparoscopic gastric bypass. Complications occurred in 8 (3.6%) patients following the intra-gastric balloon and 4 (2.5%) patients following the lap sleeve gastrectomy. There was 1 mortality (0.6%) following lap sleeve gastrectomy. The mean weight loss was 16kg (+/- 9.7) after the intra-gastric balloon and 28.7kg (+/- 14) after the Lap sleeve gastrectomy at the end of year 1. Our results from this study demonstrate that metabolic or bariatric surgical services provided by our appropriately trained and motivated team of specialists can be delivered safely in this highly selected sector of the population of Nigeria.

## Introduction

Metabolic or bariatric surgery is globally well recognized as a safe and reliable treatment option for patients seeking a surgical solution to obesity and/or obesity related diseases [1]. The multi-disciplinary team is usually the functional unit that delivers care to patients, and it is usual to have well-defined inclusion and exclusion criteria for access to such services that will be no doubt subject to a range of regional/geographic variations [2]. The impact and outcomes of metabolic or bariatric surgery remain extremely good, resulting in improvement in the quality of life of the patient, control of co-morbidities and reduction in long-term patient mortality [3]. This is due in a large part to the technical refinement of the various surgical procedures over the years leading to an overall post-operative complication rates below 3% [4]. In addition, the meticulous and streamlined peri-operative processes such as detailed pre-operative assessment and optimization, high quality intra-operative care and enhanced recovery post-operative care all work together in maintaining such outcomes in this field of surgery. There is evidence that there is a growing increase in the local population of adolescents and adults with clinical obesity, with a figure as high as 34% [5].

It is speculated that this is due to the Westernization of our pattern of living. In a recent paper, the prevalence of overweight and obesity among adults in Nigeria has been estimated at 27.6%, and 14.5% respectively [6]. Obesity has become a global health challenge and many medical groups now recognize obesity as a non-communicable disease with several pathophysiological aspects which may be physical such as diabetes, hypertension, coronary heart disease, stroke, certain kinds of cancer and pulmonary disease [7]. Psychological problems could also arise with common examples being depression, stress and anxiety [7-9]. Moreover, obesity causes societal and economic problems which may be due to repeated sicknesses from comorbidity causing increased healthcare expenditures, productivity losses due to significant impact on their health-related quality of life [7]. It is rather paradoxical in that there is still a large media and political focus on malnutrition within our local population, while obesity on the other hand is gradually becoming of public health concern with a marked increase in public health costs, increased burden on the ailing health systems and its effect on health-related quality of life [6]. There is a palpable absence of studies focusing on any aspect of metabolic or bariatric surgery in the West African region, with most papers originating from other regions of the continent [3,10,11]. This fact is not that much of a surprise considering there are only a limited number of specialized centers offering metabolic or bariatric surgery services in this region. As compared to North Africa where hundreds to thousands of procedures are regularly performed annually [12]. This study set out to review the patient clinical data, treatment outcomes and overall experience following metabolic or bariatric surgery performed by a single consultant bariatric surgeon over a 10-year period in Lagos, Nigeria.

## Methods

**Study design and study settings:** metabolic or bariatric surgical services were offered to any patient who fits the selection criteria for treatment and who demonstrated a good grasp of the knowledge and understanding of the procedure, commits to be followed up for a reasonable period after surgery (usually 2-3 years) and is medically and psychological fit. The surgical units where patients had their surgical procedures performed were two unconnected private surgical facilities. In the first 4 years after the startup and launch of the service, patients´ procedures were carried out at the Lagoon Hospital Apapa, during the subsequent 6 years, procedures were carried out at the specialist surgical units of Euracare hospital. Both hospitals are within the city of Lagos, the commercial capital city of Nigeria. This is a retrospective study based on the clinical records and captured data of all patients who underwent a surgical procedure (balloon insertion, sleeve gastrectomy and gastric bypass) from January 2012 to June 2022.

**Study population:** our services are available to obese patients who access our clinics either as a direct self-referral or a cross referral from the endocrinologist or cardiologist. Patients are usually selected onto one or more of our weight loss programs following a detailed initial meeting in our combined clinic that has present consultant surgeon and the specialist bariatric nurse. The International Federation for the Surgery of Obesity and Metabolic Disorders (IFSO) guidelines are our reference point for patient selection. Informed consent usually occurs after combined verbal conversations and patient reflection of written leaflets.

**Data collection:** the information on sociodemographic parameters, comorbidities, body mass index (BMI), post-operative complications, conversion, and post-operative 30-day morbidity and mortality were all captured real-time and prospectively in both written and digital formats. All patients were routinely discussed in our multi-disciplinary bariatric team meeting. Overweight and obesity were ranked according to the World Health Organization classification; overweight was defined as a BMI between 25 and 29-9 kg/m^2^; BMI between 30.00 and 34.99kg/m^2^ is stated as obesity class I, a BMI between 35.00 and 39.99kg/m^2^ as obesity class II and BMI >40.00kg/m^2^ as obesity class III. Type 2 diabetes mellitus was defined as a serum HbAlc > 6.5% or fasting glucose > 7 mmol/L at two occasions, or a known diagnosis of type II diabetes mellitus on treatment. Hypertension was defined as a blood pressure above 140/90mmHg. Hypercholesterolemia was defined as LDL cholesterol > 4.1 mmol/L, total cholesterol > 6.2 mmol/L, and triglycerides > 2.3 mmol/L. Obstructive sleep apnoea (OSA) was confirmed in cases where an overnight sleep polysomnography test resulted in an Apnea-Hypopnea Index (AHI) of > 5 events per hour. Operative weight was defined as the patient's weight measured closest to the time of surgery. Complications included any eventuality that resulted in deviation from the standard outcome pathway. Statistical analysis was performed using the Package for the Social Sciences (SPSS) 22.0 (SPSS Inc., Chicago, IL) software program for the data analysis. Descriptive results included frequencies, mean ± standard deviation and range, while the statistical evaluations were performed using student T-test. The significance level was set at p < 0.05.

**Metabolic or bariatric surgical interventions:** gastric balloon procedures and protocols, both intragastric balloon brands used in our clinic are the adjustable Spatz 3 and Apollo endo surgery Orbera balloon systems. The patient preparation starts 2 days before insertion. The day case balloon insertion procedures were performed by routine upper gastrointestinal endoscopy under local anesthetic throat spray and intravenous conscious sedation. The balloon is typically filled with between 500-650ml of methylene blue saline. Laparoscopic gastric sleeve procedure and protocols, All the procedures were performed on a bariatric operating table with the aid of a foot plate and legs apart accessories (Maquet). Our standard operative procedure in performing the gastric sleeve starts with the use of the verres needle at palmers point for the pneumo-peritoneum. Once the peritoneal cavity is inspected and no contra-indication to proceed was established the remaining ports were placed and liver lifted using the Nathanson system (Mediflex). Dissection was performed with either Ligasure Maryland or Harmonic scalpel. Cutting and stapling were done by either the EndoGIA or Echelon flex. A 34Fr oro-gastric tube was our standard for both the sleeve stapling calibration and the staple line methylene blue leak test. We routinely focused on staple line hemostasis just before the methylene blue leak test by ensuring the systolic blood pressure was temporarily elevated to 140-150 mmHg. Any bleeding detected is usually dealt with by applying ligaclips and/or suturing. Gastric bypass procedure and protocols. Our preference for the gastric bypass procedure was the one anastomosis gastric bypass (OAGB). The biliopancreatic limb was as a standard made 200cm long [13]. Patient's early post-operative journey was in accordance with the enhanced recovery pathway [14]. Patients were usually discharged the following day after a satisfactory clearance from the dietation combined with normal day 1 blood work. Post discharge drugs included laxatives, clexane and simple analgesics.

**Ethical consideration:** patients agree and sign a combined informed consent for the surgical procedure and data collection at the very onset pre-operation. There was no ethical committee approval required for this case series study.

## Results

**General characteristics of the study population:** between January 2012 to June 2022, a total of 381 consecutive bariatric interventions were performed in my practice. There were 220 patients (57%) who had intragastric balloon, 157 patients (41%) who had laparoscopic sleeve gastrectomy and 4 patients who had one anastomosis gastric bypass (OAGB). Patient demographics and patient comorbitity are shown in Table 1 and Table 2 respectively. In Table 3 the changes in weight and BMI are shown following intervention

**Table 1:** patient demographics

Age in years (mean ± SD)	41.4 ± 9.1 (13-75)
**Co-morbidity**	
Yes	136 (35%)
No	245 (65%)
Admitting weight (kg)	114.± 24.3 (63.8 - 216)
BMI at admission (mean±SD)	42.4 ±8.7 (24.2 - 84)
All comers post-procedural weight loss one year (kg) (mean±SD)	20.7 ±10.9 (6.1 - 78)
**Weight class**	
Overweight	17 (4%)
Class I	56 (14%)
Class II	92 (24%)
Class III	216 (56%)

**Table 2:** types of co-morbidities seen in 169 cases

Co-morbidity	Number of patients (%)
Hypertension	99 (26%)
Diabetes	33 (8.7%)
OSA	16 (4.2%)
Knee osteoarthritis	10 (2.6%)
Infertility	8 (2.1%)
Hypercholesterolemia	3 (0.8%)
OSA: obstructive sleep apnea	

**Table 3:** characteristics of patients who had a gastric balloon versus sleeve gastrectomy

	Balloon (220 cases)	Sleeve (157 cases)	P-value
Age (years)	41.4±9.0 (13 - 63)	41.5±9.3 (16 - 75)	0.91
**Sex**			
Male	40	25	0.82
Female	180	132	
Weight at presentation (kg)	116.2±27.4 (63 - 216)	128±21 (78 - 189)	0.01
Body mass index at presentation (kg/m^2^)	40.1±9.7 (27 - 84)	44.8±7.4 (28 - 69)	0.04
Co-morbidity	40/220	93/157	0.001
Weight lost after one year (kg)	16.0±9.7 (6-69)	28.7±14.0 (9 - 73.8)	0.001
Body mass index drop after one year (kg/m^2^)	5.8±3.8 (-1.6 to -25.6)	10.2±5.7 (-2.5 to -25.2_)	0.01

### Procedure related complications

**Gastric balloon patients:** a total of 220 balloon interventions were carried out during this period. The outline of the complications in this group is shown in Table 4. In this cohort there was a serious complication in 1 patient (0.4%) who suffered a linear tear to the gastro-esophageal junction at the time of insertion that required an immediate surgical repair (Table 4). The patient stayed on admission for 6 days post-surgery and had no long-term side effects. There were no deaths within the balloon cohort.

**Table 4:** type and number of complications following gastric balloon insertion

Procedure	Complication	Number of patients
Gastric balloon	Balloon intolerance	6
Gastric balloon	Spontaneous passage of balloon	3
Gastric balloon	Gastroesophageal Junction the injury	1
Gastric balloon	Leakage	1

**Lap sleeve gastrectomy and OAGB patients:** there were serious complications in 4 (2.5%) patients while there was 1 (0.6%) patient death within the 30-day post-operative period (Table 5). The one post-operative death occurred as a result of multi-organ failure (MOF) secondary to an early hemolytic blood transfusion reaction in one of the two post-operative bleed cases mentioned above. Both post-operative bleeds occurred within the first 24 hours of the procedure. In both instances the 2 patients underwent urgent re-laparoscopy and peritoneal washout. In one of the cases the bleeding spot was detected and sutured while in the second patient the bleeding had ceased and could not be accurately identified at re-laparoscopy. This latter patient unfortunately suffered multi organ failure (MOF) following her blood transfusion at died 14 days following the primary procedure. Both patients with peritoneal sepsis secondary to a staple line leak presented within 12 days of the primary surgery. The first patient re-presented on the fifth day post-surgery with left upper quadrant pain (LUQ) and a fever. Urgent abdominopelvic computed tomography scan confirmed a gastric staple line leak with a contained collection with pockets of gas restricted to the left upper quadrant. This patient had a previous open splenectomy following a road traffic accident many years prior. Initial intravenous (IV) resuscitation with fluids and antibiotic was swiftly followed by urgent re-laparoscopy and peritoneal washout.

**Table 5:** the management and outcomes of complications after the lap sleeve gastrectomy

Procedure	Complication	Re-operation	Mortality
Sleeve gastrectomy	Gastric leak with peritonitis	Repeat laparoscopy + washout and peritoneal drainage with temporary luminal stenting	No
Sleeve gastrectomy	Small persistent proximal staple line leak	Repeat laparoscopy, ogd and long-term percutaneous cavity drainage	No
Sleeve gastrectomy	Staple line bleeding	Repeat laparoscopy and washout.	Yes
Sleeve gastrectomy	Staple line bleeding	Repeat laparoscopy and hemostatic control with sutures	No

A 5 mm staple line leak in the upper stomach area was detected and an on-table endoscopy failed to pick up any gastric tube kink or staple line stricture. The leak was sutured in 2 layers and two drains left in the vicinity along with an NG tube and a feeding jejunostomy was performed. The immediate post-operative recovery was uneventful. However, in this instance, the acute leak transitioned into a chronic fistula was successfully managed by long-term cavity percutaneous drainage and irrigation. Cavitograms where helpful here and complete closure occurred at 5 months post initial procedure. The patient with the second staple leak presented on day 12 post-surgery. The presentation in this instance was less dramatic, mostly LUQ pains with very little features of sepsis. Computed tomography scan confirmed a contained leak in the LUQ, this was initially managed by IV antibiotics and percutaneous drainage. Computed tomography oral contrast studies confirmed a persistent upper gastric staple line leak that in this case was successfully managed by a combination of re-laparoscopy washout with further drainage combined with aid of an 8-week removable (covered) oesophago-gastric stent.

## Discussion

This study investigated the patient demographics, clinical interventions and outcomes following metabolic or bariatric surgery performed by a single bariatric surgeon in Lagos. Obesity and diseases linked to obesity remain a global concern and there are reports revealing that between 1975 and 2016 the prevalence of obesity has nearly tripled worldwide. In 2016 there were 1.9 billion adults with overweight; of these, 650 million adults were suffering from obesity [15]. Gender, age, living in an urban area, reduced physical activity, illiteracy, high income and diet have been implicated as risk factors for obesity in Nigeria [16,17]. In the last three decades, there has been a fast rise in the number of fast-food outlets in most urban communities visited by largely upper and middle class citizens with an increase in consumption of energy dense foods filled with saturated fat [6]. In addition to this there is a reduced level of physical activity combined with alcohol consumption tied to urbanization, industrialization, globalization, and lifestyle changes leading to nutrition transition which results in distortion or extinction of indigenous food and food habits and increasing adoption of the western diet which is energy dense [6].

The link between the rising prevalence in obesity and type II diabetes mellitus is undisputed and the WHO estimates that the highest increase in the global incidence of type II DM will be detected in low to middle-income countries and regions, in particular, sub-Saharan Africa (SSA) [18]. The re-classification by certain international organizations of obesity as a stand-alone chronic disease has been necessary in an effort promote an awareness that obesity is not simply caused by affluence and sedentary living, to also highlight that the life-threatening risk that occurs with obesity [7]. The additional advantage of this re-classification to draw the attention of healthcare planners, health service funders, local and national governments that obesity is epidemic within the society, affects all ages and is largely a treatable disease. Metabolic or bariatric surgery is widely accepted as a safe and efficacious surgical treatment option in the global fight against obesity, resulting in not just weight loss but also other health indicators leading to improved quality of life and overall health parameters [19,20]. A recent IFSO 7^th^ registry report 2022 gives an up-to-date picture of what is happening globally in terms of total reported surgical procedures, regional variations, patient demographics and outcome measures. A major challenge in Sub-Saharan Africa (SSA) remains the availability of specialists surgical centers that are committed to evidence-based surgical management for eligible patients.

**Figure 1:**
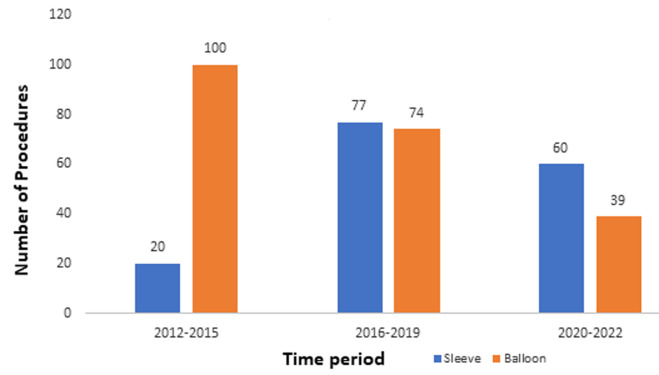
graph of the comparative (Balloon vs Sleeve/OAGB) distribution of cases over 10 years divided into 3 distinct time block periods

Interestingly, the awareness and as a result the request for metabolic or bariatric surgery services continues to grow in our region. The first choice of where patients opt to seek treatment is unclear. There is no way of accurately identifying the total numbers of patients seeking such services overseas (health/medical tourism) but anecdotal and personal experiences suggest large volumes of patients are still travelling along old and well established post colonial routes in the pursuit of surgical treatment of their obesity. Our unit was developed with the main purpose of giving patients the option of accessing metabolic or bariatric surgical care closer to home in our region of West Africa. The unit is led by an experienced bariatric surgeon and functions as a modern day bariatric/metabolic multi disciplinary team made up of several clinical and non-clinical personnel whose sole interest is the provision of the best evidence-based care available in the region. Understandably the uptake was quite slow and remains low in general, however, our year-on-year increase in numbers since 2016 remain consistent (Figure 1). Our results highlight that in our unit the average age of patients seeking treatment was 41.4years with females accounting for 82% of all cases. This is similar to other studies done in Africa [3,11]. This also supports the data that female obesity in sub-Saharan Africa far outnumbers that of men which is in contrast with the developed world where the prevalence of obesity in men is similar to or even exceeds that in the female population. The mean BMI at presentation was 42.4 kg/m^2^ with 56.7% of the patients being class III obese which is similar to presentations in other Montpellier Business School (MBS) centers [2,11].

**Figure 2:**
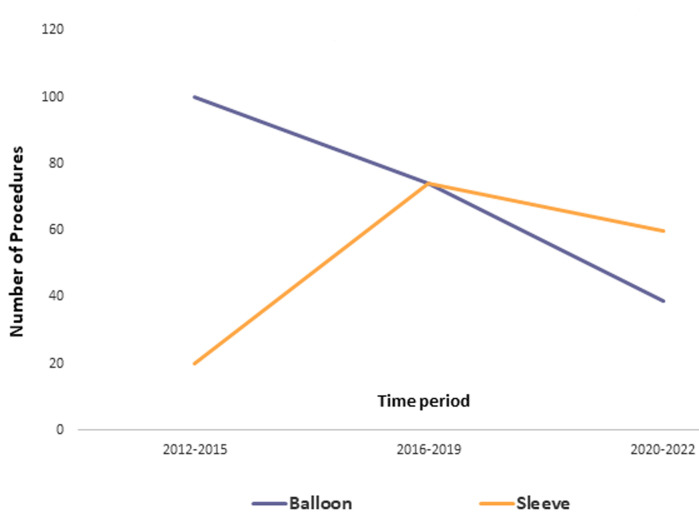
the change in numbers of sleeve and balloon procedures over the period of 10 years is shown in this slide split into 3 distinct time block

The mean total weight loss after one year was 16kg for the balloon and 28.7kg for patients who had sleeve gastrectomy. Also, the mean drop in BMI following the balloon and sleeve gastrectomy were 5.8kg/m^2^ and 10.2kg/m^2^ respectively. Our findings over the 10 years as it relates to patient surgical procedure choice suggest that as more and more patient stories emerged as either mouth to mouth or via social media testimonials patients were less anxious or fearful in choosing the operative weight loss procedure over the non-operative gastric balloon procedure (Figure 2). This is a welcome finding as it suggests that benefits of metabolic or bariatric surgery is gradually gaining acceptance within our communities. There were balloon complications in a total of 11 patients (5%). The 1 major complication was a serious and rare complication of gastro-esophageal (GOJ) injury at the time of the balloon insertion. The error was detected immediately and treated appropriately by surgery that involved emergency Laparoscopy and a laparoscopic repair of the injury was feasible and successful in managing this complication. The patient made a speedy and uneventful post-operative recovery being discharged home on post-operative day 4 on liquid diet. In the surgical group of patients, a total of four patients required to return to theatre for re-laparoscopy because of staple line bleeding (two patients) and leak (two patients) making the overall complication rate 2.5% which is lower than the 8% reported by Sofianos *et al*. [3]. There was one (0.6%) mortality within the first 30 post-operative days which is higher than the 0.2% rate as reported by Diamantis *et al*. [4].

This death occurred from multi-organ failure as direct consequence of a blood transfusion reaction. The main limitation of this case series is the limited patient numbers, a single surgeons data and a relatively short 1 year post surgery follow-up information. The setting up and delivering MBS in Nigeria has been both challenging and a rewarding endeavor. We have witnessed the slow but convincing acceptance by not just the people in our communities as to the positive impact that metabolic or bariatric surgery can result in, but also our fellow healthcare workers particularly the cardiologists and endocrinologists. Our team continues to grow and deliver improved care for our patients. Literature review and interactions with colleagues within the West African region and sub-Saharan in general reveals that despite centers emerging randomly in various areas there remains very few published articles outlining the regions outcomes and experiences with the delivery of MBS [3,11].

## Conclusion

This case review aimed at analyzing our data in order to establish more objectively the results of this novel and new specialist MBS service in our region. Despite the limited overall patient numbers and understandable regional obstacles to the growth of MBS in the region, this report demonstrates that evidence based and safe MBS services are available in Lagos, Nigeria. It is imperative that clinicians and clinical institutions keen on getting involved in the growth and expansion in the delivery of MBS services in our region as by product of demand and supply forces, invest heavily and deeply in not just clinical excellence but also minimum standard infrastructure in order to avoid surges in patient complications rates and therefore any potential negative impact on the growth and wider acceptance of MBS in our region. We are cautiously optimistic for the future growth and expansion of MBS services in the region.

### What is known about this topic

- Metabolic or bariatric surgery has gained huge popularity globally as an intervention to tackle the epidemic of obesity with extremely good impact and outcomes;
- The adoption and spread of this service in sub-Saharan Africa is extremely limited with at best 2 very small studies into outcomes;
- There is significant investment in terms of infrastructure and manpower required in the set up and delivery of such tertiary services.

### What this study adds

- This would be the largest cohort of patient data and outcomes so far presented and published in the region.
